# Facile Fabrication of Conductive Graphene/Polyurethane Foam Composite and Its Application on Flexible Piezo-Resistive Sensors

**DOI:** 10.3390/polym11081289

**Published:** 2019-08-01

**Authors:** Weibing Zhong, Xincheng Ding, Weixin Li, Chengyandan Shen, Ashish Yadav, Yuanli Chen, Mingze Bao, Haiqing Jiang, Dong Wang

**Affiliations:** 1 College of Chemistry, Chemical Engineering and Biotechnology, Donghua University, Shanghai 201620, China; 2 Hubei Key Laboratory of Advanced Textile Materials & Application, Wuhan Textile University, Wuhan 430200, China

**Keywords:** reduced graphene oxide, polyurethane foam, flexible electronics, pressure sensing

## Abstract

Flexible pressure sensors have attracted tremendous research interests due to their wide applications in wearable electronics and smart robots. The easy-to-obtain fabrication and stable signal output are meaningful for the practical application of flexible pressure sensors. The graphene/polyurethane foam composites are prepared to develop a convenient method for piezo-resistive devices with simple structure and outstanding sensing performance. Graphene oxide was prepared through the modified Hummers method. Polyurethane foam was kept to soak in the obtained graphene oxide aqueous solution and then dried. After that, reduced graphene oxide/polyurethane composite foam has been fabricated under air phase reduction by hydrazine hydrate vapor. The chemical components and micro morphologies of the prepared samples have been observed by using FT-IR and scanning electron microscopy (SEM). The results predicted that the graphene is tightly adhered to the bare surface of the pores. The pressure sensing performance has been also evaluated by measuring the sensitivity, durability, and response time. The results indicate that the value of sensitivity under the range of 0–6 kPa and 6–25 kPa are 0.17 kPa^−1^ and 0.005 kPa^−1^, respectively. Cycling stability test has been performed 30 times under three varying pressures. The signal output just exhibits slight fluctuations, which represents the good cycling stability of the pressure sensor. At the same stage, the response time of loading and unloading of 20 g weight turned out to be about 300 ms. These consequences showed the superiority of graphene/polyurethane composite foam while applied in piezo-resistive devices including wide sensitive pressure range, high sensitivity, outstanding durability, and fast response.

## 1. Introduction

With the rapid development of the information technology, flexible pressure sensors have been extremely concerned due to their potential in human machine interface, flexible robots, wearing electronic equipment, and flexible electronic skin [1,2,3,4,5,6,7,8,9,10,11,12,13,14,15]. Normally, flexible pressure sensors are divided into three types: capacitive, piezoelectric, and piezo-resistive. Among them, piezo-resistive sensor is the most favorite because of its simple manufacturing techniques and easily collecting signal output [2,16,17,18,19]. 

A typical method to prepare the flexible pressure sensors is to construct a composite with elastomer material as the matrix and the conductive filling materials. The external force induced compression deformation would lead to the rearrangement of the conductive fillings, which finally changed the resistance of the elastic composite [13,20,21,22,23]. Generally, the piezo-resistive materials were mainly fabricated with conductive particles filled with elastomer composites. The deformation of the elastomer would force the conductive particles to form another electrical conductive network. Besides, thin conductive film such as carbon nanotubes (CNTs) and graphene were laminated on the elastomer film to improve the connecting efficiency of the conductive components. However, the deformations of these solid materials required overcoming strong energy to move the molecular chains, which seriously restricts the sensitivity of the manufactured piezo-resistive materials [24,25,26]. Obviously, substrate with lower Young’s modulus possesses stronger deformation ability which significantly contributes the sensitivity. Polyurethane (PU) foam, which is an elastic material with large amount of through-pore structures and exhibiting low Young’s modulus and high resilience, is an ideal candidate for high performance substrate for piezo-resistive sensors.

Graphene is a two-dimensional (2D) carbon material which has excellent physical properties [16,19,27]. It is a honeycomb shaped 2D planar crystal which is composed of 6 sp^2^ hybrid carbon atoms connected by σ bond. As reported [28,29,30,31], graphene has exhibited excellent physical properties. For instance, specific surface area is as high as 2600 m^2^g^−1^, ultralow areal density of 0.77 mg/m^2^, the optical transmittance can be as high as 98%, high electron mobility and thermal conductivity of 2.5 × 10^5^ cm^2^/Vs and 5000 Wm^−1^K^−1^, respectively. Therefore, graphene shows great potential in many fields.

In this paper, a convenient method of preparing the flexible piezo-resistive sensors is proposed. the reduce graphene oxide (rGO)/PU foam composite are fabricated via soaking the GO aqueous solution with pure PU foam and then reduced with hydrazine hydrate vapors. The chemical structure as well as the micro morphologies have been observed. Except that, the pressure sensing performance such as sensitivity, recognition ability to different pressures, cycling stability and response time have been systematically investigated. Due to the simple fabrication method, easily obtained raw materials and controllable preparation conditions, the composite materials can be manufactured with high reproducibility and very low cost. Except that, the chemically inert of the rGO enables it with good reusability over time as long as they were stored and used within the moderate environment. 

## 2. Experimental

### 2.1. Materials

Fourier infrared tester (Tensor 27, Bruker, Ettlingen, Germany) and scanning electronic microscope (6510LV, JEOL, Tokyo, Japan) are applied for chemical structure and micro morphologies measurements. Electrochemical workstation (PGSTAT302N, Metrohm Autolab, Utrecht, Netherlands) and RLC digital electric bridge (TH2818, Tonghui, Changzhou, China) are used to detect and record the resistance of the composites foam under varying pressure. Digital display force gauge (Mark-10, ESM301, MARK-TEN, New York, NY, USA) and relative tension and compression testing bench used for applying the pressures. The microcrystal graphite used is purchased from Qingdao Jiaodong graphite Co., Ltd., Qingdao, China. The other chemicals used in the experiments are obtained from Sinopharm Chemical Reagent Co., Ltd., Shanghai, China. The deionized water is self-made in our lab.

### 2.2. The Preparation of Graphite

Modified Hummers method is used to synthesize the well delaminated GO. The oxidizing agents were concentrated with sulfuric acid and potassium permanganate. The acid and inorganic salts were removed from GO by centrifugation and dialysis. The purified GO aqueous solution was then configured to a fixed concentration for subsequent use.

### 2.3. Preparation of the Reduced Graphene Oxid/Polyurethane (rGO/PU) Foam Flexible Conductive Materials

**Figure 1:**
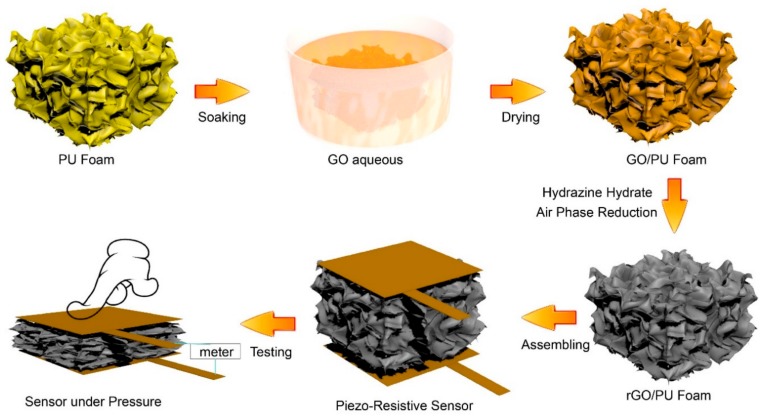
Schematic figure of Preparation and assembling of the reduced graphene oxide/polyurethane (rGO/PU) foam piezo-resistive sensor.

As Figure 1 shows, the polyurethane (PU) foam was sliced into a fixed size of 40 × 40 × 30 mm^3^. They were pre-cleaned in the alcohol and then dried in oven at 60 °C for 2 h. After that, the PU foam were dipped into the GO aqueous solution with concentration of 3 mg/mL for 15 min. During this period, the foam was constantly squeezed in order to adsorb as much GO aqueous solution as possible. Then, the PU foam was transferred into vacuum oven at 55 °C for 4 h. After the drying process, the GO/PU foam was hanged in a sealed bottle with 8 mL hydrazine hydrate with concentration of 85 wt.%. The bottle was heated with oil bath with temperature of 100 °C for 90 min. The rGO/PU foam composite was obtained after three times cleaning and drying. The digital camera photographs of the pure PU foam and the rGO/PU composite foam was displayed in Figure S1.

### 2.4. Characterization and Methods

The quality of the GO and rGO were measured and analyzed by using X-ray diffraction (XRD). The chemical structure as well as the micro morphologies were observed with Fourier Transform infrared spectroscopy (FTIR) and Scanning electronic microscope (SEM). After that, the piezo-resistive sensor was assembled by adhering the copper electrodes to the two surfaces with 4 × 4 cm^2^ of the conductive sponges. The pressure sensing performance such as sensitivity, recognition ability to different pressures, cycling stability, and response time were systematically investigated by using the electrochemical workstation, RLC digital electric bridge, digital display force gauge, and relative tension and compression testing bench.

## 3. Results and Discussions

### 3.1. X-ray Diffraction (XRD) Spectrum of Graphene Oxide (GO) and Reduced Graphene Oxide (rGO) 

**Figure 2:**
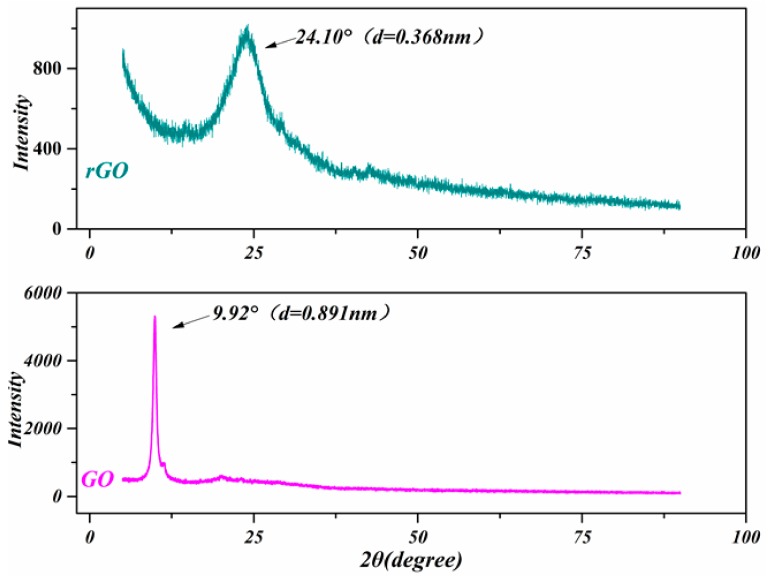
The X-ray diffraction (XRD) spectrum of graphene oxide (GO) and reduced graphene oxide (rGO) prepared with modified Hummers method.

Figure 2 presents the XRD spectrum of the prepared GO and rGO. As can be seen, both spectra show an upward single peak. Being different, the peak position of the GO and rGO were 9.92° and 24.10°. Relying on the Bragg’s law, the crystals distance of GO and rGO are observed 0.891 nm and 0.368 nm respectively. Due to the violent oxidation reaction, large amounts of oxygen-containing groups occur in layers of the graphite during the preparation, and bigger inter planar spacing forms. These oxygen-containing groups are progressively removed during the reduction, so that the layer spacing decreases to 0.368 nm. It is closed to the layer spacing of natural flake graphite, which is 0.34 nm. This is the main reason to showing XRD results. The GO and rGO were prepared accordingly previous reported literatures [32,33]. 

### 3.2. Chemical Structure Analysis

**Figure 3:**
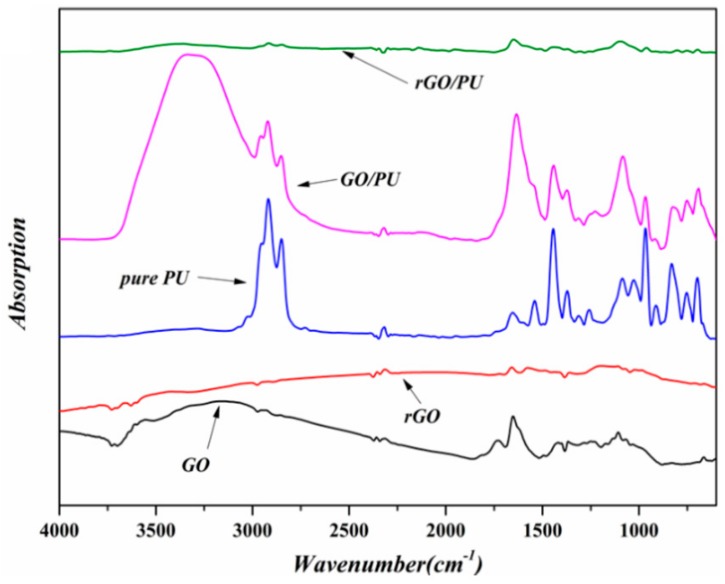
Fourier-transform infrared (FT-IR) of the graphene oxide (GO), reduced graphene oxide (rGO), pure polyurethane (PU), graphene oxide/polyurethane (GO/PU) and reduced graphene oxide/polyurethane (rGO/PU) composite foam.

Figure 3 shows the FTIR spectra of GO and rGO, blank PU foam obtained using Fourier Transform infrared spectroscopy. Infrared characteristic absorption peak of GO is shown in above figure. The stronger wide peak for free hydroxyl stretching resonance absorption at 3430 cm^−1^. The tip of peak for telescopic resonance absorption peak of carboxyl-carbon-oxygen double bond in GO at 1725 cm^−1^. The bending resonance absorption peak of C–OH is at 1630 cm^−1^. Carbon oxide carbon epoxy bond absorption peak is at 1110 cm^−1^. This indicates that the prepared GO have free hydroxyl and carboxyl groups and these groups are reduced by hydrazine hydrate vapor. The rGO has a little hydroxyl and epoxy groups. Comparing the change of the characteristic absorption peak in 5 infrared absorption curves, it could be known that after the compounding of PU foam and rGO, the peak of PU, itself has been weakened and the absorbing peaks of GO have occurred. In addition, the characteristic absorption peaks in the infrared spectra of rGO/PU foam are similar with the pure rGO. It can be inferred that the hydrazine hydrate vapor reduced rGO completely wrapped the PU foam because the specific absorption peaks of PU foam completely disappeared.

### 3.3. Micro Morphology

**Figure 4:**
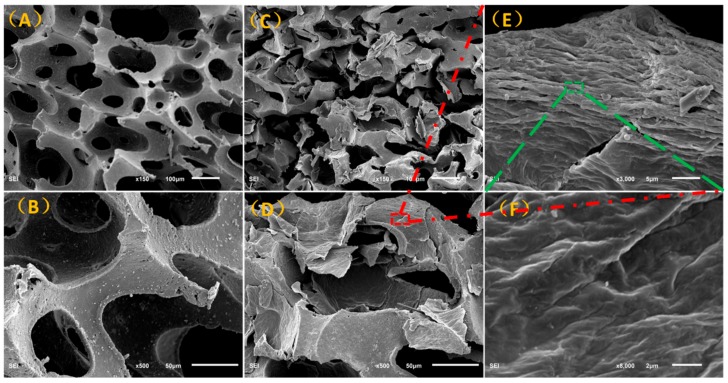
Scanning electron microscopy (SEM) images of pure polyurethane (PU) foam (**A**,**B**) and reduced graphene oxide/polyurethane (rGO/PU) foam (**C**–**F**) with varying magnifications.

Figure 4 shows the SEM images of a pure PU foam and rGO/PU foam, where Figure 4A, and B are images of pure PU foam at different magnifications, Figure 4C–F are rGO/PU foam at different magnifications. It can be seen from the images that the pores of the PU foam are regular, evenly distributed, and the surface is rough, which helps for the adhesion of GO.

From the Figure 4C–F, the rGO is sufficiently arranged on the wall of the foam pores. The surface of the rGO/PU foam exhibits lots of wrinkles and burrs. The directional arrangements of the rGO can be easily observed though the SEM image with high magnification (Figure 4E). The through-pore structure would be compressed while the external pressure was loaded on the composite foam. The wall of the foam pores that consisted of direactional arrangement rGOs would contact to each other forming electron transmission pathways. The wrinkles and burrs becomes “micro-switches” to adjust the contact area and quantity that dominated the equivalent circuit of the composite foam. As a result, the resistance would change rapidly when the rGO were rearranged under the pressure. This process was illustrated in Figure S2.

### 3.4. Sensitivity

**Figure 5:**
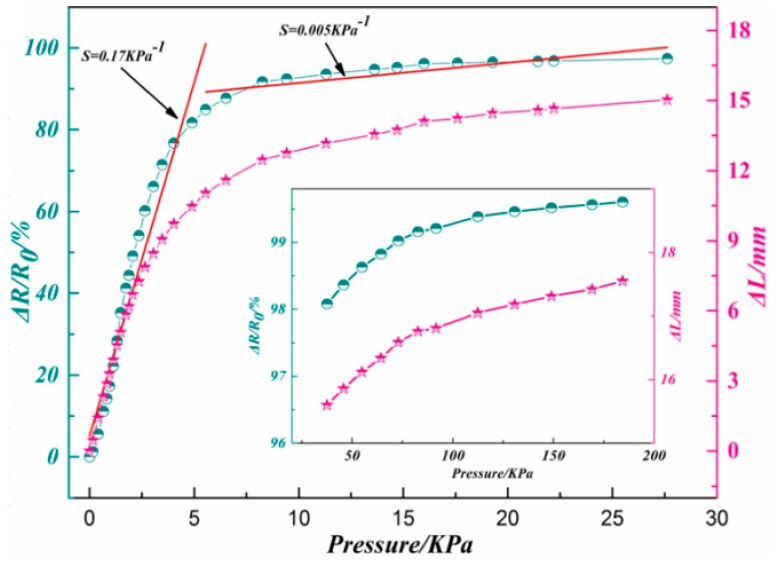
Relative resistance changes and compression amounts of reduced graphene oxide/polyurethane (rGO/PU) composite foam under pressure bellow 30 kPa.

To evaluate the sensing performance of the prepared rGO/PU foam composite, a series of different pressures were loaded on the assembled piezo-resistive sensor. The resistance and the compression amount were recorded. The original electrical resistance of the assembled piezo-resistive sensor was about 7.35 kΩ. Figure 5 represents the calculated relative resistance changes (Δ*R/R*_0_) and compression amount (Δ*L*) curves to the varying pressure. As can be seen, the relative resistance change shows similar trend with the compression. The relative resistance change and compression amount increased rapidly both in the pressure range of 0–6 kPa and then the rate of rise gradually decreases in the larger pressure range of 6–25 kPa. This may be caused by the variation of the pore structure during the compression process, which result in the different change rate of the contact area of the graphene sheets on the pore walls.

(1) $$S=\frac{\partial (\Delta R/R_{0})}{\partial P}$$

(2) $$\Delta R=R-R_{0}$$

where the *R* refers to the resistance under the pressure *p* and the *R*_0_ refers to the original resistance of the piezo-resistive device. 

According to the definition of equation of sensitivity (Equations (1) and (2)) [8,9] the Δ*R/R*_0_ curve can be divided into two linear sections and fitted. The slope of the two linear fitted line can be considered as the sensitivity in that pressure range. From the figure, the value of sensitivity under the range of 0–6 kPa and 6–25 kPa are 0.17 kPa^−1^ and 0.005 kPa^−1^, respectively.

### 3.5. Pressure Discrimination

**Figure 6:**
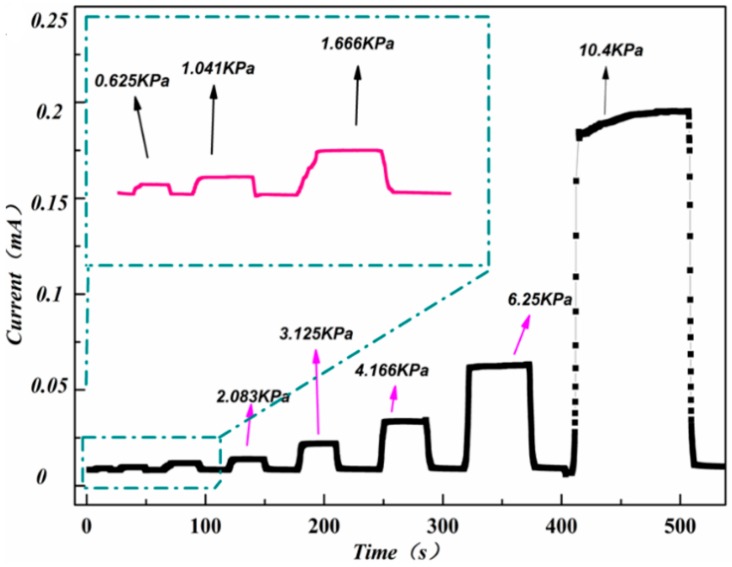
Real-time current response of reduced graphene oxide/polyurethane (rGO/PU) composite foam under different pressure.

Figure 6 presents the real-time current response under constant voltage supply of 0.5 V when varying pressure (ranging from 0.625 kPa to 10.4 kPa) were loaded on the rGO/PU foam composite. As can be seen, lots of current stages existed in the very stable based line. What is worth noting is that the stage and the baseline are almost parallel to the horizontal axis. This indicates the high signal to noise ratio of the piezo-resistive sensor. The height of the current stage increased while the applied pressure increased which means the resistance of the device decreased. What is more, the different stable current stages induced by various pressures shows the excellent pressure discrimination of the piezo-resistive sensor which is practical useful to the application scenario requiring specific pressure value.

### 3.6. Cycling Stability

**Figure 7:**
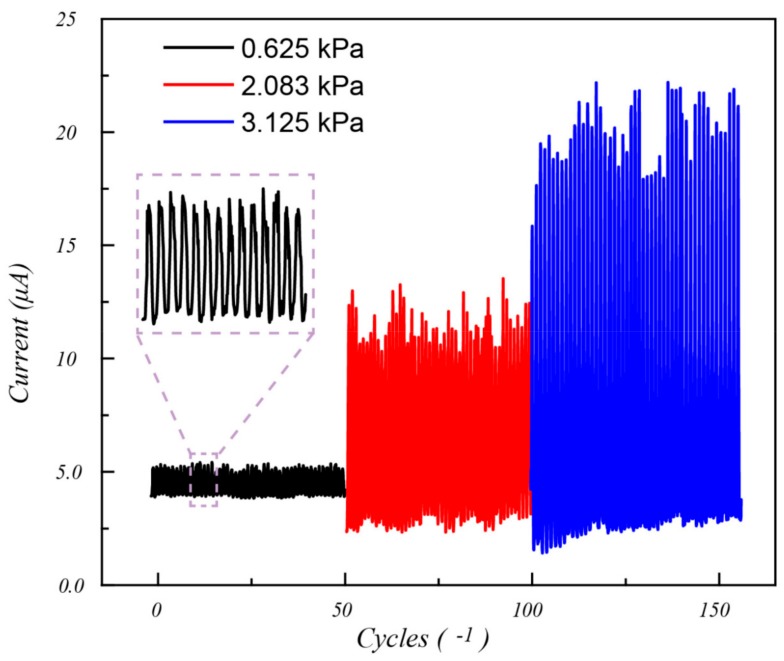
The 50 times cycling stability of the prepared reduced graphene oxide/polyurethane (rGO/PU) foam composite sensor under 0.625, 2.083 and 3.125 kPa.

In order to measure the cycling stability of the sensor, the different pressure of 0.625 kPa, 2.083 kPa, and 3.125 kPa were repeatedly applied and removed on the rGO/PU foam composite sensor for 30 times with time interval of 0.5 s. The results were displayed in Figure 7. As can be seen, there are several slight fluctuations for the cycling stability testing of each pressure. This may be caused by the little difference of the contacting area even under the same pressure. Actually, the rigid rGO plane fulfilled in the PU foam pore could contribute to the cycling stability by limiting the compression deformation trajectory. 

In addition, comparing with different pressures, there is a little offset of the current change while removing the pressure. This may be caused by the inherent creep and stress relaxation properties of the PU foam. In other word, the PU foam structure changed themselves to resist the applied pressure but need a long time to recover, which is expressed as the offset of baseline.

### 3.7. Response Time

**Figure 8:**
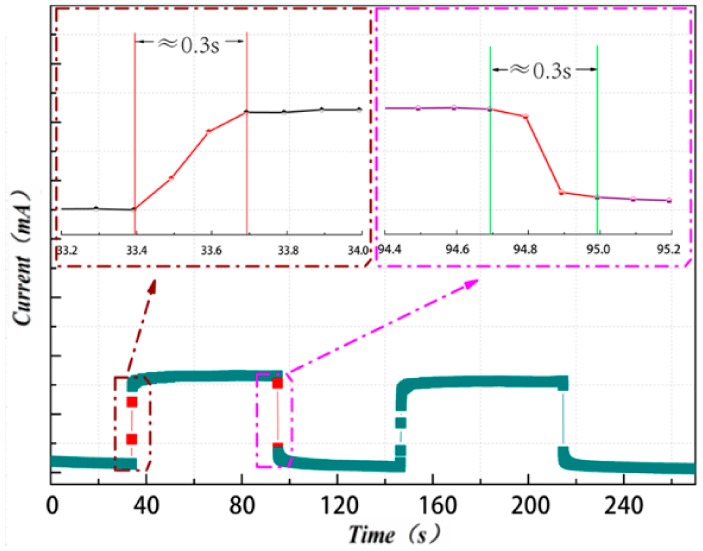
The response time and the recovery time of the reduced graphene oxide/polyurethane (rGO/PU) composite foam sensor.

The response time was tested by loading and unloading a fixed pressure with 20 g weight on the piezo-resistive sensor with fast speed. The response time (τ) could match the formula τ = τ_m_ + τ_s_. Among them, τ_m_ is the time that the rGO/PU composite foam induct the pressure change when adding or removing the force; τ_s_ is the time which start from the rGO/PU composite foam inducting the pressure to the electrical parameters finishing the change. As τ_m_ is small and cannot be detected accurately, the value of τ_s_ is used to represent the completely response time of rGO/PU in this study. From Figure 8, we can find that the τ_s_ is about 0.3 s while using the 20 g weight as the pressure applying object. 

## 4. Conclusions

In this present research, the graphene/polyurethane foam composites are prepared to develop a convenient method for piezo-resistive devices with simple structure and outstanding sensing performance. The chemical components and micro morphologies have been observed by using FT-IR and SEM. The results predicted that the graphene is tightly adhered to the bare surface of the pores. The pressure sensing performance has been also evaluated by measuring the sensitivity, durability, and response time. The results indicate that the value of sensitivity under the range of 0–6 kPa and 6–25 kPa are 0.17 kPa^−1^ and 0.005 kPa^−1^, respectively. Cycling stability test has been performed 30 times under three varying pressures. The signal output just exhibits slight fluctuations, which represents the good cycling stability of the pressure sensor. At the same stage, the response time of loading and unloading of 20 g weight were turn out about 300 ms. These consequences showed the superiorities of graphene/polyurethane composite foam while applied in piezo-resistive devices including wide sensitive pressure range, high sensitivity, outstanding durability, and fast response.

## Supplementary Materials

The following are available online at https://www.mdpi.com/2073-4360/11/8/1289/s1, Figure S1: The digital camera photographs of (a) pure PU foam, (b) prepared rGO/PU composite foam, (c) Digital display force gauge and relative tension and compression testing bench and (d) Electrochemical workstation; Figure S2: The pressure sensing mechanism of the rGO/PU composite foam sensor with pore structures and switchable rGO wrinkles and burrs.
