# Supplementary material for: Facile Fabrication of Conductive Graphene/Polyurethane Foam Composite and Its Application on Flexible Piezo-Resistive Sensors
Source: Polymers (Basel). 2019 Aug 1;11(8):1289. doi: 10.3390/polym11081289 (PMC6722995; doi:10.3390/polym11081289)
Supplement: Supplementary file 1 [file polymers-11-01289-s001.pdf]

Supporting Information

Facile Fabrication of Conductive Graphene/Polyurethane Foam Composite and its Application on Flexible Piezo-Resistive Sensors

Weibing Zhong[1], Xincheng Ding[2], Weixin Li[2], Chengyandan Shen[2], Ashish Yadav[2], Yuanli Chen[2], Mingze Bao[2], Haiqing Jiang[2*], Dong Wang[1,2*]

1. College of Chemistry, Chemical Engineering and Biotechnology，Donghua University，Shanghai，201620，China；
2. Hubei Key Laboratory of Advanced Textile Materials & Application, Wuhan Textile University, Wuhan, 430200, China）

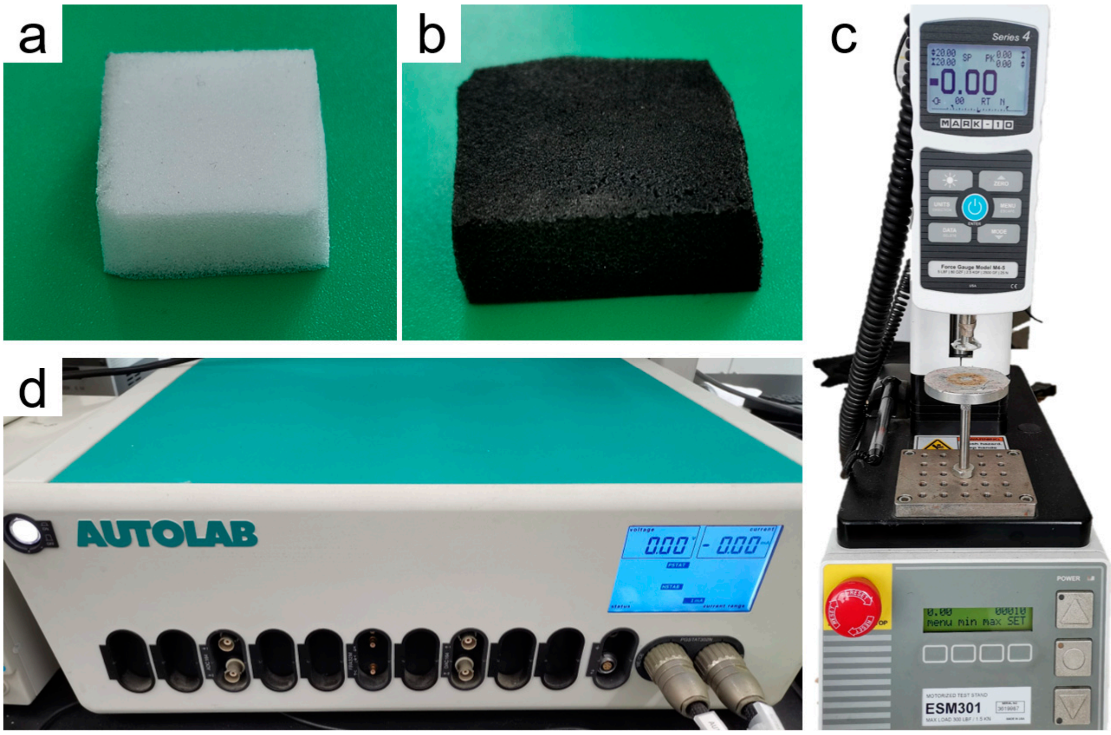

Figure S1. The digital camera photographs of (a) pure PU foam, (b) prepared rGO/PU composite foam, (c) Digital display force gauge and relative tension and compression testing bench and (d) Electrochemical workstation.

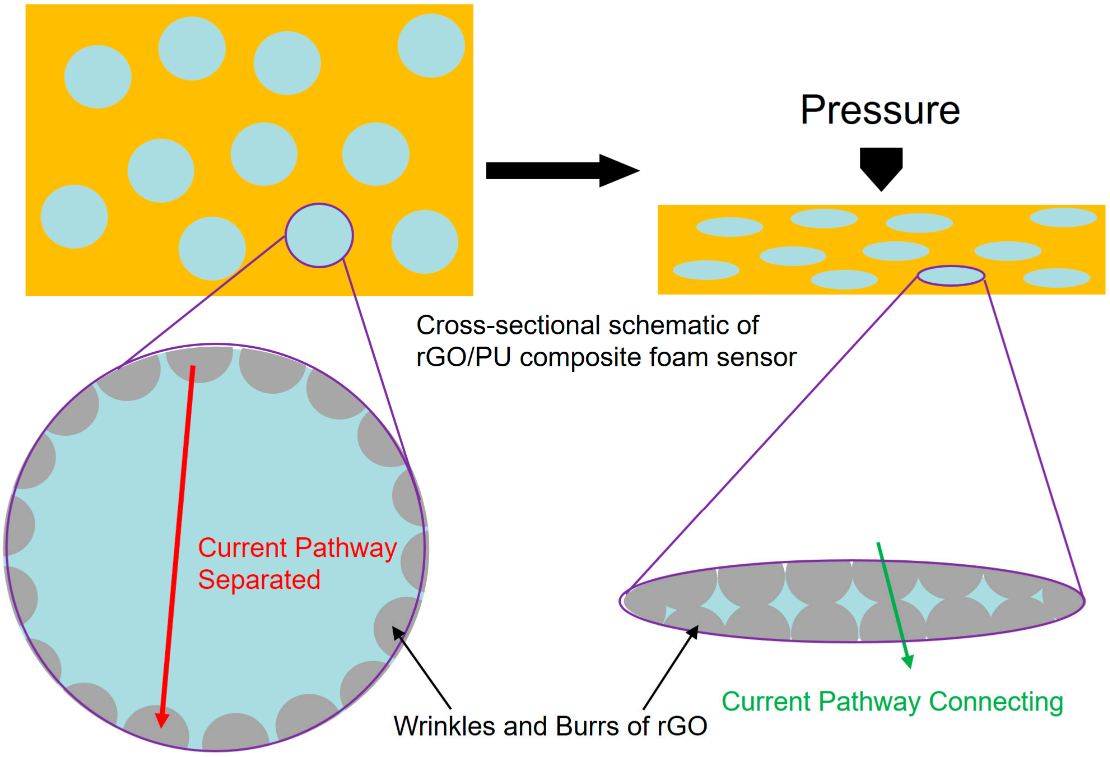

Figure S2. The pressure sensing mechanism of the rGO/PU composite foam sensor with pore structures and switchable rGO wrinkles and burrs.
